# Root-TransUNet enables high-throughput phenotyping of *Arabidopsis thaliana* roots as a parameter in *Heterodera schachtii* parasitism

**DOI:** 10.3389/fpls.2026.1939674

**Published:** 2026-09-04

**Authors:** Jie Zhou, Victor Hugo Moura de Souza, Lei Ju, Sebastian Eves-van den Akker, Ji Zhou, Olaf Prosper Kranse

**Affiliations:** 1 College of Engineering, Plant Phenomics Research Centre, Academy for Advanced Interdisciplinary Studies, Nanjing Agricultural University, Nanjing, China; 2 Crop Science Centre, Department of Plant Sciences, University of Cambridge, Cambridge, United Kingdom; 3 Cambridge Crop Research, National Institute of Agricultural Botany (Niab), Cambridge, United Kingdom

**Keywords:** *Arabidopsis thaliana* MAGIC population, computer vision, *Heterodera schachtii*, plant-nematode interactions, root morphology

## Abstract

**Introduction:**

Plant parasitism by sedentary plant-parasitic nematodes is a dynamic and continuously evolving process, accompanied by profound remodelling of host root system architecture across distinct infection stages. However, the physiology and anisotropic growth of Arabidopsis thaliana roots under Heterodera schachtii infection, together with complex lateral root proliferation and increasingly dense, overlapping morphology, pose substantial challenges for accurate image segmentation.

**Methods:**

Here, we introduce Root-TransUNet, an optimised segmentation architecture that extends TransUNet by incorporating a composite loss function to improve boundary precision and structural continuity, as well as dual-stage strip-pooling (SP) modules to enhance elongated and directional root features. These adaptations address the unique morphological complexity of the infected root system. Additionally, we integrated Root-TransUNet into a high-throughput phenotyping pipeline and applied it to an existing dataset of ~120,000 images of 362 A. thaliana MAGIC recombinant inbred lines collected over several months of infection. By extracting root system architecture traits, including root surface area and estimated root volume across infection stages, we enabled stage-specific association analyses between host root growth and nematode performance across these genotypes.

**Results:**

Root-TransUNet achieved strong segmentation performance, demonstrating improved structural continuity and boundary precision compared with widely used CNN- and Transformer-based baselines, including UNet++. Stage-specific analyses revealed that the relationship between host root traits and nematode performance changed as infection progressed. During establishment, nematode number was largely independent of initial root size and varied strongly among genotypes, whereas during the reproductive phase (10-30 dpi), greater root expansion coincided with reduced estimated nematode volume accumulation. Notably, nematode burden was largely independent of host root size before infection, indicating that root quantity was generally not a limiting factor for infection in this experiment.

**Discussion:**

These results demonstrate that Root-TransUNet can robustly segment infected root systems across a wide range of nematode infection densities, providing a scalable image-analysis framework for studying plant-parasitic nematode parasitism in combination with host root phenotyping.

## 1 Introduction

Plant-parasitic nematodes (PPNs) pose a serious threat to global food security, causing estimated annual losses of ~$173 billion (Dutta et al., 2019), and cyst nematodes (*Heterodera* spp.) are amongst the most destructive. The cyst nematode *Heterodera schachtii* has been widely adopted as a tractable model because it readily infects *Arabidopsis thaliana*, a model plant species, in tissue culture systems (Baum et al., 2000). Following destructive intercellular migration, *H. schachtii* second-stage juveniles (J2) select a single cell within the vascular cylinder, which is induced to form a syncytium through the fusion of hundreds of neighbouring cells. Concurrent with syncytium establishment, the female nematode becomes sedentary and feeds non-destructively from this persistent metabolic sink for the remainder of her lifecycle (several weeks) (Hofmann and Grundler, 2006). This parasitic process is accompanied by profound remodelling of host root system architecture, including induction of secondary lateral roots near infection sites (Sijmons et al., 1991), with potential cascading consequences for whole-plant growth.

Capturing the dynamic interaction between host root system architecture and nematode parasitism on a large scale poses a significant methodological challenge. Traditional root phenotyping often relies on final, destructive assays, such as manual harvesting of root systems to determine fresh and dry weights or to quantify total root biomass (Kuijken et al., 2015; Paez-Garcia et al., 2015). These end-point methods capture only a single time point and are labour-intensive, limiting scalability and their applicability across large populations and successive stages of infection. Although automated image analysis has enabled the extraction of static and dynamic traits from large time-series datasets in other plant phenotyping areas (Sun et al., 2022, Sun et al., 2026), applying similar dynamic phenotyping analyses to root-nematode interaction remains challenging. Semi-automated, heuristic-based, phenotyping of roots and nematodes in the *H. schachtii*:*A. thaliana* system was achieved using open-source imaging hardware combined with thresholding-based segmentation (Kranse et al., 2022). However, that method was limited to a single pre-infection time point for root quantification and became progressively unreliable as infection progressed and root morphology grew more complex.

To address the limitations of traditional image analysis methods, vision-based deep learning techniques, particularly U-Net and its variants, have driven substantial progress in automated plant root phenotyping. U-Net is an encoder-decoder convolutional neural network designed for pixel-wise image segmentation. Its encoder progressively extracts contextual features, whilst its decoder restores spatial resolution; skip-connections between corresponding encoder and decoder stages combine high-level semantic information with fine spatial detail, enabling precise delineation of small or complex structures (Ronneberger et al., 2015). These architectures are widely deployed for robust root segmentation across diverse species, enabling fully automated analysis (Narisetti et al., 2021), precise segmentation of root networks (Seidenthal et al., 2022), and the extraction of complex root architectures from noisy soil backgrounds (Yasrab et al., 2019; Smith et al., 2020). In parallel, deep learning frameworks have supported the detection and developmental staging of nematodes in plants (Akintayo et al., 2018; Chen et al., 2019; Saikai et al., 2024) (Wei et al. unpublished). However, the segmentation of *A. thaliana* roots specifically under *H. schachtii* infection poses unique challenges that standard convolutional neural network (CNN) architectures (O’Shea and Nash, 2015) struggle to overcome. Roots of *A. thaliana* are inherently slender and develop dense, overlapping, branching structures during the course of infection. In such demanding scenarios, conventional CNNs frequently produce fragmented segmentations, unable to maintain the continuity of fine lateral roots, accurately delineate ambiguous boundaries at root-nematode contact points, or precisely represent the highly anisotropic geometry of infected root systems.

Here, we present Root-TransUNet, a hybrid segmentation architecture that combines a U-Net-based encoder-decoder with vision transformer (ViT)-based global contextual modelling and dual-stage strip-pooling modules to enhance the representation of root structures. Implemented within a high-throughput phenotyping pipeline based on a custom 3D-printed imaging platform (Wei et al. unpublished), Root-TransUNet enables the extraction of root system architecture traits, including root surface area, as well as the estimation of root volume across defined infection timepoints. When integrated with nematode development measures (Wei et al. unpublished), this framework enabled stage-specific association analyses between host root system architecture traits and nematode performance across 362 genotypes. These analyses showed that host-parasite associations were not constant across infection stages. During establishment, the initial root surface area showed only a weak, genotype-dependent relationship with nematode numbers, whereas during the reproductive phase (10–30 dpi), estimated root volume expansion became negatively associated with estimated nematode volume accumulation. Thus, larger or actively expanding roots did not simply translate into a higher parasite burden; rather, host growth and parasite development shifted towards an antagonistic relationship as infection progressed, whilst total nematode burden remained broadly consistent across host root size classes.

## 2 Materials and methods

### 2.1 High-throughput phenotyping and image acquisition

Images used for root segmentation and phenotyping were obtained from an existing repository: https://www.ebi.ac.uk/biostudies/bioimages/studies/S-BIAD2402 which is published under CC BY 4.0 license (https://creativecommons.org/licenses/by/4.0/legalcode). This dataset contains a time series of RGB images acquired via a custom built, 3D-printed automated imaging system, described in detail from Wei et al. unpublished). Specifically, 527 *Arabidopsis thaliana* Multiparent Advanced Generation Inter-Cross (MAGIC) (Kover et al., 2009) recombinant inbred lines were grown, each with approximately 20 biological replicates. In that study, plants were grown individually in 5-cm petri dishes containing standard KNOP medium supplemented with 0.8% (w/v) Daishin agar and Gamborg Vitamin B5 solution, and inoculated with approximately 80 second-stage juveniles (J2) of the sugar-beet cyst nematode *Heterodera schachtii* per plant. Imaging was carried out semi-automatically using a custom built platform equipped with Raspberry Pi HQ cameras (12.3 megapixels) with a 16 mm Telephoto Lens (RASPBERRY-PI). Over an 86-day period, approximately 400,000 images were captured documenting both nematode development and dynamic changes in root system architecture. From this repository, a quality-controlled subset of approximately 120,000 images from 362 lines was used for root segmentation and phenotypic analysis in this study.

### 2.2 Image preprocessing

To ensure longitudinal spatial consistency and to correct for shifts inherent in high-throughput image acquisition, raw images were registered using a fixed QR-code marker on the surface of each petri dish. QR-code regions were detected and calibrated to a common reference frame using the scale-invariant feature transform (SIFT, OpenCV V4.12) (Burger and Burge, 2022) for feature matching, combined with RANSAC-based (OpenCV V4.12) (Fischler and Bolles, 1987) homography estimation to correct geometric distortions. The petri dish region was then segmented via HSV colour thresholding and morphological operations to isolate the largest connected component. Finally, the circular Hough transform (Pei and Horng, 1995) was applied to generate a standardised circular mask for precise cropping of the petri dish (**Supplementary Figure 1**).

### 2.3 Dataset construction and image augmentation

After spatial normalisation, each petri dish image was divided into non-overlapping 512 × 512 pixel patches. Zero-padding was applied where necessary to ensure uniform input dimensions. Initially, ~100 of representative patches were manually annotated using LabelMe (Russell et al., 2008). A preliminary U-Net model (Ronneberger et al., 2015), trained on these manually curated samples, was then applied to the unlabelled patches to generate initial segmentation masks. These predictions were refined using the interactive annotation platform Napari (Chiu et al., 2022), with pixel-level corrections performed to improve boundary precision and structural continuity. The annotated dataset encompassed diverse infection stages and root densities. The data were divided into training, validation and test sets in an 8:1:1 ratio. To enhance generalisation, offline augmentations, including horizontal and vertical flipping, were applied to approximately 1,250 images before training (**Supplementary Table 1**).

### 2.4 The architecture of Root-TransUNet

To address the challenges posed by *A. thaliana* root systems in petri dish images, specifically their slenderness, high anisotropy and complex branching, we developed Root-TransUNet, a novel segmentation architecture that combined global contextual modelling by transformers with the precise spatial localisation of U-Net. We chose TransUNet as the baseline, not as a simple substitute but because its hybrid CNN-transformer design captured both long‑range dependencies and fine‑grained morphological detail, which are critical for resolving complex root topologies (Chen et al., 2021).

**Figure 1:**
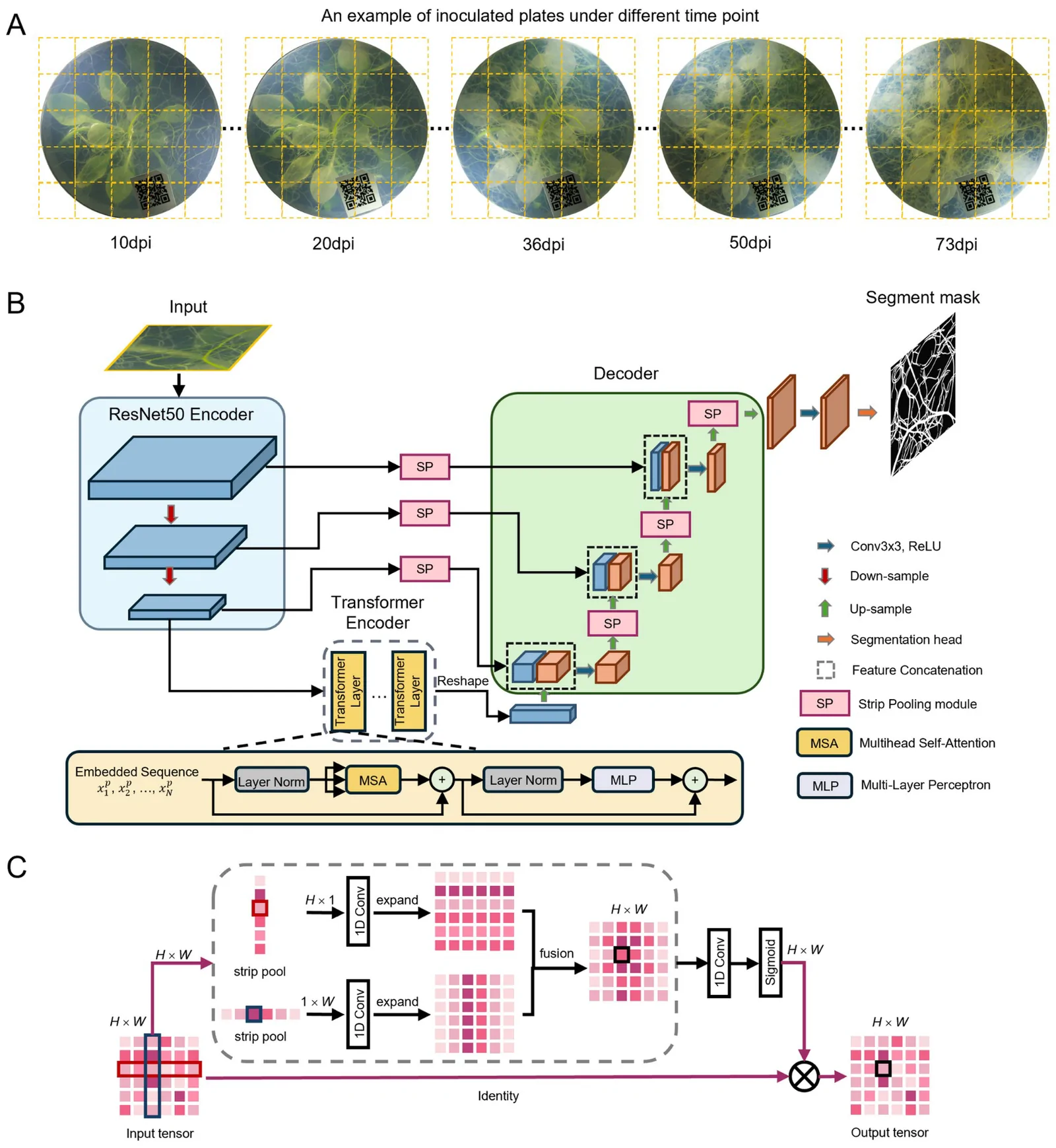
Overview of the root segmentation and phenotyping integration framework. **(A)** Representative petri dish images of *A. thaliana* root under *H. schachtii* infection across different days post inoculation (dpi). Full-plate RGB images were divided into non-overlapping 512 x 512 patches (yellow prior to model training, whereas whole-image inference was performed using overlapping sliding windows followed by probability-map fusion). **(B)** Architecture of the proposed Root-TransUNet model. The network is built upon the TransUNet backbone, combining convolutional encoding with transformer-based global context modelling. To better capture the elongated and continuous morphology of *Arabidopsis* roots, strip pooling (SP) modules are integrated into both skip connections and decoder blocks as a dual-stage structural enhancement mechanism. **(C)** Structure of the strip pooling (SP) module. The module performs directional pooling operations to aggregate long-range contextual information along horizontal and vertical axes. By enhancing anisotropic feature responses, the SP module strengthens the representation of slender root segments whilst suppressing irregular background noise, thereby improving structural continuity and connectivity in segmentation outputs.

*Arabidopsis* roots often measured less than two pixels in diameter in the high‑resolution petri dish images; direct downsampling would therefore severely degrade morphological information. To avoid this, we adopted a patch‑based preprocessing strategy. Full‑plate RGB images of *A. thaliana* infected with *H. schachtii* from 10 to 73 days post‑inoculation (dpi) were cropped into non‑overlapping patches (**Figure 1A**), preserving fine spatial resolution whilst retaining computational efficiency.

Root-TransUNet used a hybrid encoder-decoder architecture (Ronneberger et al., 2015). A pre-trained ResNet-50 (He et al., 2015) functioned as the backbone and extracted local edge and texture features efficiently. Shallow encoder features were forwarded to the decoder via skip connections to support high-precision boundary reconstruction. Conventional U-Net-based architectures rely predominantly on convolutional operations to extract local and multiscale features. Their ability to model long-range relationships can therefore be limited, particularly when spatially separated root segments form part of the same continuous structure. To address this limitation, Root-TransUNet incorporated a ViT module at the deepest encoder stage (Dosovitskiy et al., 2020). Convolutional feature maps were flattened into sequences and linearly projected into a hidden space, where a multi-head self-attention mechanism captured global associations (**Figure 1B**). This design is intended to capture the overall topological structure of the root system and to address the locality limitations inherent in purely convolutional encoders.

Root-TransUNet also differs from conventional U-Net approaches through the a dual-stage strip pooling (SP) strategy (**Figures 1B, C**) (Hou et al., 2020). Unlike standard square pooling kernels, which can incorporate background noise from the agar medium when extracting elongated root features, strip pooling aggregates contextual information along the horizontal and vertical axes. The strategy comprised two complementary modules: (1) pre-fusion feature purification, deployed before the encoder-decoder skip connections. In this stage, narrow kernels pool features along the vertical and horizontal axes. This stage is designed to suppress texture clutter arising from the agar matrix in low-level features and to emphasise the skeletal structure of both primary and lateral roots. (2) post-fusion connectivity enforcement, embedded within the cascaded upsampler decoder. Here, strip pooling was applied after feature concatenation and convolution. By aggregating contextual information across spatial dimensions, this stage aims to reinforce the global connectivity of slender root structures and to bridge local discontinuities caused by occlusion, low contrast or signal attenuation.

For model optimisation, we employed a composite loss function combining the focal Tversky loss (Abraham and Khan, 2019) with the soft-clDice loss (Shit et al., 2021) to address extreme foreground sparsity and the strong requirement for structural continuity. The focal Tversky loss weighted false negatives more heavily than false positives (*β* = 0.7, *α* = 0.3), thereby improving the recall of hard-to-detect targets, such as fine lateral roots. Meanwhile, the soft-clDice loss assessed topological consistency through soft skeletonization of the predicted probability maps and imposed explicit constraints on structural errors, including breaks, artifacts, and missing branches.

### 2.5 Training strategy

For the Root-TransUNet model, online augmentations (random rotation, contrast variation, random noise, simulated occlusion, and foreground-specific intensity perturbation) were applied to enhance model generalisation. The model was trained for 300 epochs using Stochastic Gradient Descent optimiser (Bottou, 2010) with an initial learning rate of 0.01, a momentum of 0.9, a weight decay of 0.0001, and a batch size of 8. To ensure stable convergence, a polynomial learning rate decay strategy was employed, in which the learning rate at each iteration was scaled by ${\left(1-\frac{i}{N}\right)}^{0.9}$, where *i* is the current iteration step and *N* is the total number of max iterations.

Despite the moderate dataset size, the use of ImageNet-pretrained weights for the ResNet-50 backbone, combined with the extensive online augmentation strategy described above, mitigated overfitting risks, as confirmed by stable validation loss convergence. The checkpoint achieving the lowest validation loss was retained for all subsequent inference and evaluation.

### 2.6 Whole-image inference and mask reconstruction

After model training, Root-TransUNet was applied to full petri-dish images using a sliding-window inference strategy. As direct downsampling of the full-resolution images would obscure thin lateral roots, each pre-processed petri-dish crop was divided into 512 × 512 pixel windows for local prediction. During whole-image inference, adjacent windows were sampled with overlap, and the resulting probability maps were projected back to their original spatial coordinates. For pixels covered by multiple windows, the corresponding probabilities were averaged prior to thresholding to generate the final binary root mask. This probability-fusion strategy reduced boundary artefacts arising from hard patch stitching and improved the continuity of roots crossing patch boundaries. The reconstructed masks were then cropped to the original petri-dish region and used for subsequent estimation of root area and volumetric traits.

### 2.7 Detection of number of nematodes

To associate host root traits with a measure of parasitism, we required a method to quantify nematode parasitism most accurately. Not every life stage is reliably detectable by the computer vision model. The moment at which the model best predicts the actual number of nematodes is identified using a data-driven algorithm building upon the deep-learning pipeline described in our concurrent work (Wei et al., unpublished). If plotted as a time-series, this point would show as a plateau line (the model counts the same number of nematodes between time points for a given time window). The point at which the detection is stable (i.e. the most reliable), we refer to as the ‘estimated nematode count’ throughout this paper for ease of reading. Progression from the plateau to an increasing slope would indicate the emergence of the next generation.

The algorithm proceeded in three steps: (1) Stable-run detection: the number of nematodes per plate over time was segmented into consecutive intervals exhibiting minimal variation. Specifically, periods during which nematode abundance remained effectively constant across successive imaging sessions. (2) Candidate stable intervals extraction: each candidate interval was required to span at least 5 consecutive imaging time points. This criterion ensured that observed population stability was corroborated across multiple independent measurement sessions, rather than reflecting a single-observation artefact. (3) Temporal prioritization: candidate intervals were sorted chronologically, and the earliest such interval was designated the first-generation plateau (**Supplementary Figure 2**).

The representative plateau trait was defined as the median number of nematodes within the identified plateau window. The median was selected to ensure robustness against occasional imaging artefacts and to avoid making distributional assumptions for small observation windows (**Supplementary Figure 3**).

### 2.8 Volumetric estimations

Nematode length (*L*) and width (*W*) were obtained from the deep-learning pipeline (Wei et al., unpublished). Using these morphological traits, we estimated nematode volume by approximating the individual as a prolate spheroid (a lemon-shaped ellipsoid), with volume calculated as:


$$V_{nema}=\frac{4}{3}\pi \left(\frac{L}{2}\right)\left(\frac{W}{2}\right)\left(\frac{W}{2}\right)$$

Simultaneously, the root system architecture segmented by Root-TransUNet was modelled using a skeletonization-based cylindrical slicing approach. To account for the varying thickness across different root orders, an Euclidean Distance Transform was applied to the binary root masks. For each pixel *i* along the extracted root skeleton, the distance to the nearest background was defined as the local radius (*r_i_*). Each skeletal pixel was then treated as the center of an infinitesimal cylinder of height equal to one pixel. To obtain the physical volume, a spatial scaling factor *s* (physical length per pixel, e.g., mm/pixel) was applied. The total physical volume of the root system was integrated as follows:


$$V_{root}=\left(\sum_{i=1}^{n}\pi r_{i}^{2}\times s^{3}\right)$$

We note that both *V_nema_* and *V_root_* represent morphometric estimates derived from 2D image data, rather than direct 3D measurements, these quantities are referred to in this manuscript as volumetric estimates. Changes in volume estimate (Δ*volume*) were calculated between 10 and 30 dpi for both roots and nematodes.

### 2.9 Software implementation and statistical analysis

The Root-TransUNet model was trained using PyTorch (V2.5.1) (Paszke et al., 2019) on an NVIDIA RTX 3090 GPU, and an AMD Ryzen 9 5950X 16-Core Processor CPU, with Python (V3.9) used for model implementation. Other key open scientific libraries for statistical analysis included SciPy (V1.13.1) (Virtanen et al., 2020), NumPy (V2.0.2) (Harris et al., 2020), scikit-learn (V1.6.1) (Pedregosa et al., 2012), and pandas (V2.3.3) (McKinney, 2010).

For statistical analysis, line-level phenotypic values were estimated using best linear unbiased estimates (BLUEs) (Henderson, 1975). To ensure the statistical robustness and accuracy of the BLUEs calculations, regions quality control was applied to the initial dataset comprising 527 MAGIC lines. Replicates showing physical imaging artefacts (e.g., petri dish shifted) or biological contamination (e.g., root nodules) were excluded. Only lines with at least five valid biological replicates were retained for downstream analysis, yielding a final subset of 362 lines.

For each root and nematode trait, a linear mixed model was fitted with the MAGIC line as a fixed effect. Experimental and positional effects were included as random effects. The greenhouse blocking structure was modelled as a bench, with a tray nested within the bench and a sorted layer (Elayer) nested within the bench and tray. Inoculation-related variation was accounted for by including inoculator, jar and harvest time of nematodes as separate random effects. Models were fitted separately for each phenotype using the ‘lme4’ (V2.0-1) (Bates et al., 2015) and ‘lmerTest’ (V3.2-1) (Kuznetsova et al., 2017) packages in R package, and best linear unbiased estimators (BLUEs) for lines were extracted as estimated marginal means using the ‘emmeans’ (V2.0.3) (Russell and Piaskowski, 2026) package.

For phenotyping data visualisation, scatter plots were used to characterise population-level associations between root and nematode traits across these 362 MAGIC lines, with linear regression overlaid to quantify the direction and magnitude of each association. P-values for linear associations were calculated using simple ordinary least-squares linear regression and represent two-sided tests. To dissect line-specific variation underlying the population trend, per-line regression slopes between initial root surface area and nematode number were estimated from replicate-plate data.

For root-size classification, K-means clustering (Likas et al., 2003) was applied to BLUEs of initial root surface area to stratify the 362 genotypes into five size classes. Trait changes between 10 and 30 dpi across size classes were visualised using boxplots. The normality of trait-change distribution within each class was first assessed using the Shapiro-Wilk test (p< 0.05) (Shapiro and Wilk, 1965); combined with the qq-plots deviation from the diagonal reference line at the tail (**Supplementary Figure 5**) the assumption of normality was rejected.

Pairwise differences in median trait changes between adjacent and non-adjacent size classes were assessed with the Mann-Whitney U test (Nachar, 2008) and summarised as forest plots, with 95% bootstrap confidence intervals based on 5,000 resamples and effect sizes reported as Cliff’s delta (δ) (Macbeth and Razumiejczyk, 2011) (**Supplementary Figure 4**). Significance thresholds were defined as *** p< 0.001, ** p< 0.01, * p< 0.05, and ns (not significant). The optimum number of breakpoints to prevent overfitting was calculated as one (p< 0.05, Davies test) using piecewise_regression (v1.5.0) and the model parameters were calculated with the same package (Pilgrim, 2021).

## 3 Results

### 3.1 Ablation experiments of the root segmentation framework

**Figure 2:**
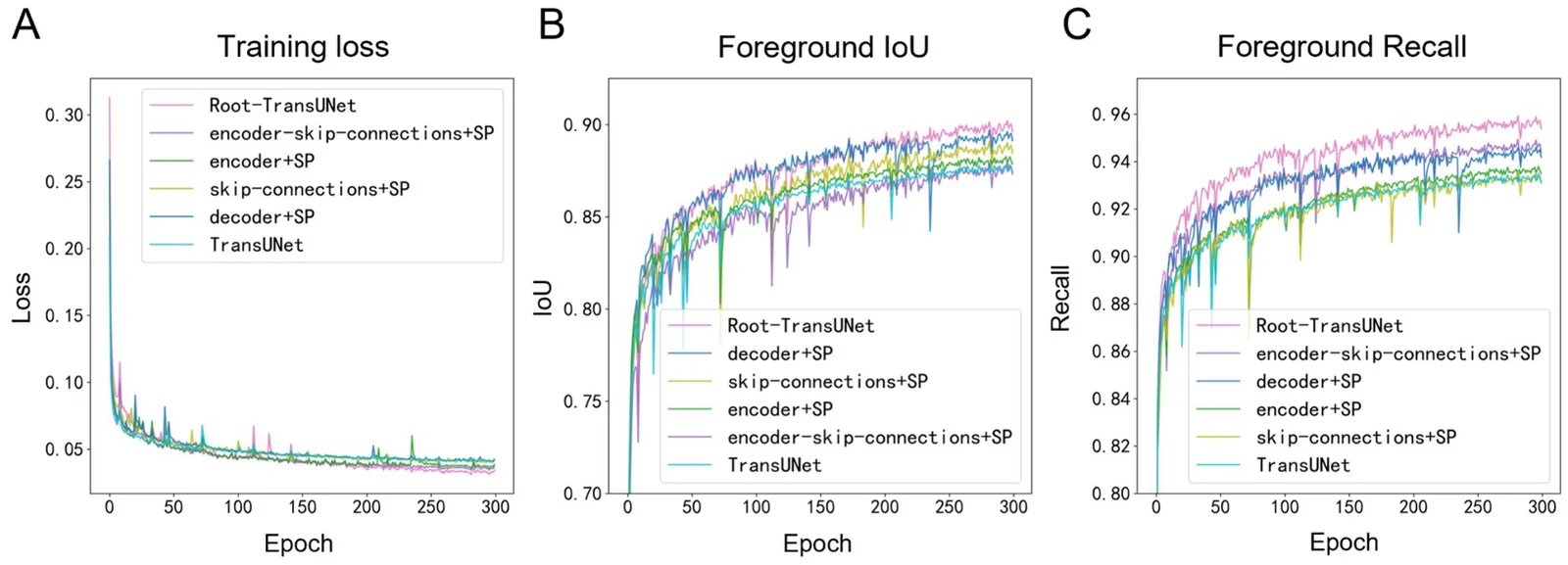
Performance comparison of Root-TransUNet in ablation experiments. Training curves of **(A)** loss, **(B)** IoU, and **(C)** recall, comparing the baseline TransUNet with variants that incorporate SP in the encoder, decoder, and skip connections.

This study presented ablation experiments on the Root-TransUNet model, with all metrics evaluated on the validation set (**Figure 2**, **Supplementary Table 1**). Before assessing the spatial placement of strip pooling (SP) modules, we first evaluated the effect of the composite loss function on the baseline TransUNet. The composite loss function improved the Dice from 0.935 to 0.937, the IoU from 0.879 to 0.884, and the precision from 0.935 to 0.950, whilst reducing Hd95 from 1.176 to 1.021 (**Supplementary Table 2**), relative to the initial loss setting. These results indicate that the composite loss function provided a more stable optimization objective for thin-root segmentation.

The integration of SP modules, particularly at multiple levels within skip connections, led to measurable improvements in optimization stability and segmentation accuracy. Specifically, SP-augmented variants exhibited smoother loss curves and higher Dice (Dice, 1945), IoU and recall than the baseline TransUNet (Dice = 0.935, IoU = 0.879, Recall = 0.930). Crucially, multi-level insertion consistently outperformed single-location alternatives: adding SP to skip connections alone (Dice = 0.940, IoU = 0.891) or to the decoder alone (Dice = 0.946, IoU = 0.897) each yielded intermediate improvements. In contrast, their combination in Root-TransUNet, i.e., SP modules in both skip-connection and the decoder, achieved the highest Dice (0.949), IoU (0.902) and Recall (0.955), underscoring the value of hierarchical, scale-aware feature fusion for recovering fine-grained anatomical structures. The convergence behaviour further confirmed this advantage: Root-TransUNet achieved the lowest final validation loss (0.031), indicating that the proposed architectural refinements enhanced convergence robustness and structural sensitivity relative to the baseline model (**Supplementary Table 3**).

### 3.2 Comparison experiments of the root segmentation algorithm

To evaluate the robustness of Root-TransUNet for root phenotyping, we compared it with U-Net (Ronneberger et al., 2015), U-Net++ (Zhou et al., 2018), DeepLabV3+ (Chen et al., 2018), and Swin-UNet (Cao et al., 2023) under identical training conditions, with final performance evaluated on the test set (**Table 1**). Root-TransUNet achieved the highest performance across most metrics. Compared with the strongest CNN baseline (U-Net++), the Dice score (Dice, 1945) increased from 0.928 to 0.949, the IoU increased from 0.869 to 0.902, and the 95th percentile of Hausdorff distance (Hd95) decreased by nearly 50% (from 1.737 to 0.868). The substantial reduction in Hd95 indicated improved boundary precision and structural continuity, both of which were critical for accurate skeleton reconstruction and topology extraction. Although Swin-UNet employed global self-attention, its performance was inferior on this task. The hybrid CNN-Transformer architecture of Root-TransUNet integrated local detail extraction with global contextual aggregation more effectively, particularly benefiting the segmentation of elongated and anisotropic root structures.

**Table 1:** Model comparison experiments.

Model	Dice↑	IoU↑	Precision↑	Recall↑	Hd95↓
SwinUNet	0.845	0.737	0.866	0.830	4.715
DeeplabV3+	0.875	0.781	0.893	0.875	3.380
UNet	0.920	0.847	0.926	0.909	2.786
UNet++	0.928	0.869	0.933	0.928	1.737
TransUNet	0.935	0.879	0.935	0.930	1.176
**Root-TransUNet**	**0.949**	**0.902**	**0.941**	**0.955**	**0.868**

Values in bold are for the model developed in this study.

**Figure 3:**
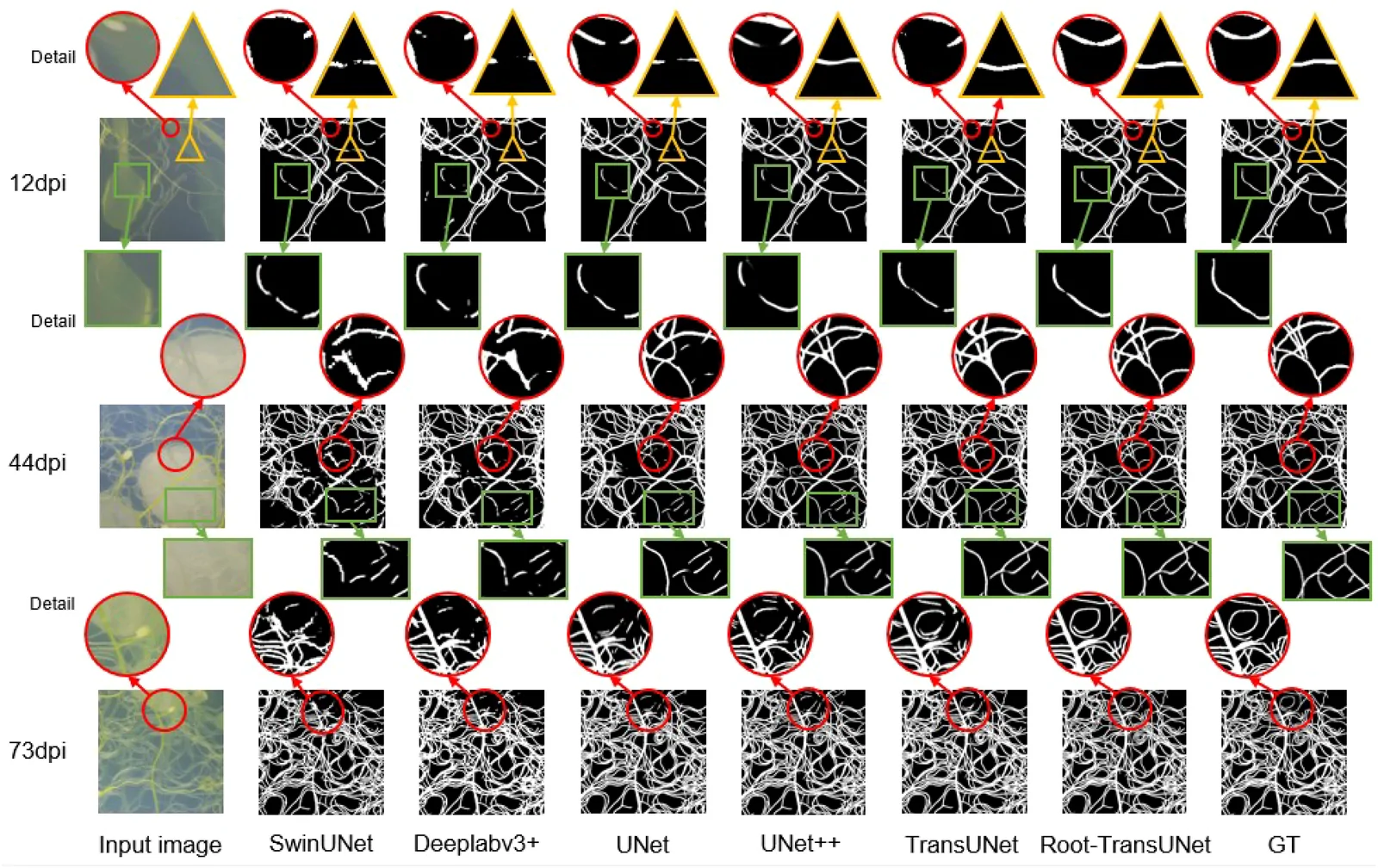
Visual quality comparison across infection stages for different models. Representative segmentation results from SwimUNet, DeepLabV3+, UNet, UNet++, TransUNet and Root-TransUNet are shown at 12, 44, and 73 dpi. Coloured markers indicate representative structural errors, including broken roots, duplicated root segments, and artificial branch discontinuities. Compared with the other models, Root-TransUNet preserved fine lateral roots more effectively as root architectures became increasingly complex.

Representative segmentation results indicated that CNN-based models commonly exhibited structural errors such as root fragmentation, omission of thin lateral roots, and misclassification of backgrounds. In contrast, Root-TransUNet maintained better axial continuity, enhanced recovery of fine roots, and reduced artificial fragmentation. Qualitative comparisons at 12, 44, and 73 dpi further illustrated consistent structural integrity as morphological complexity increased (**Figure 3**). At early stages (12 dpi), competing models occasionally fragmented emerging lateral roots. During the maturation stage (44 dpi), root breakage and missing branches became more noticeable, especially when leaves overlap with the root system. In the late stages (73 dpi), dense overlap frequently resulted in broken segments and false positives. In general, across all scenarios, Root-TransUNet mostly maintained stable structural continuity and demonstrated robust generalisation under infection-induced architectural variations.

### 3.3 Phase-specific associations between root growth and nematode performance across *A. thaliana* MAGIC population

**Figure 4:**
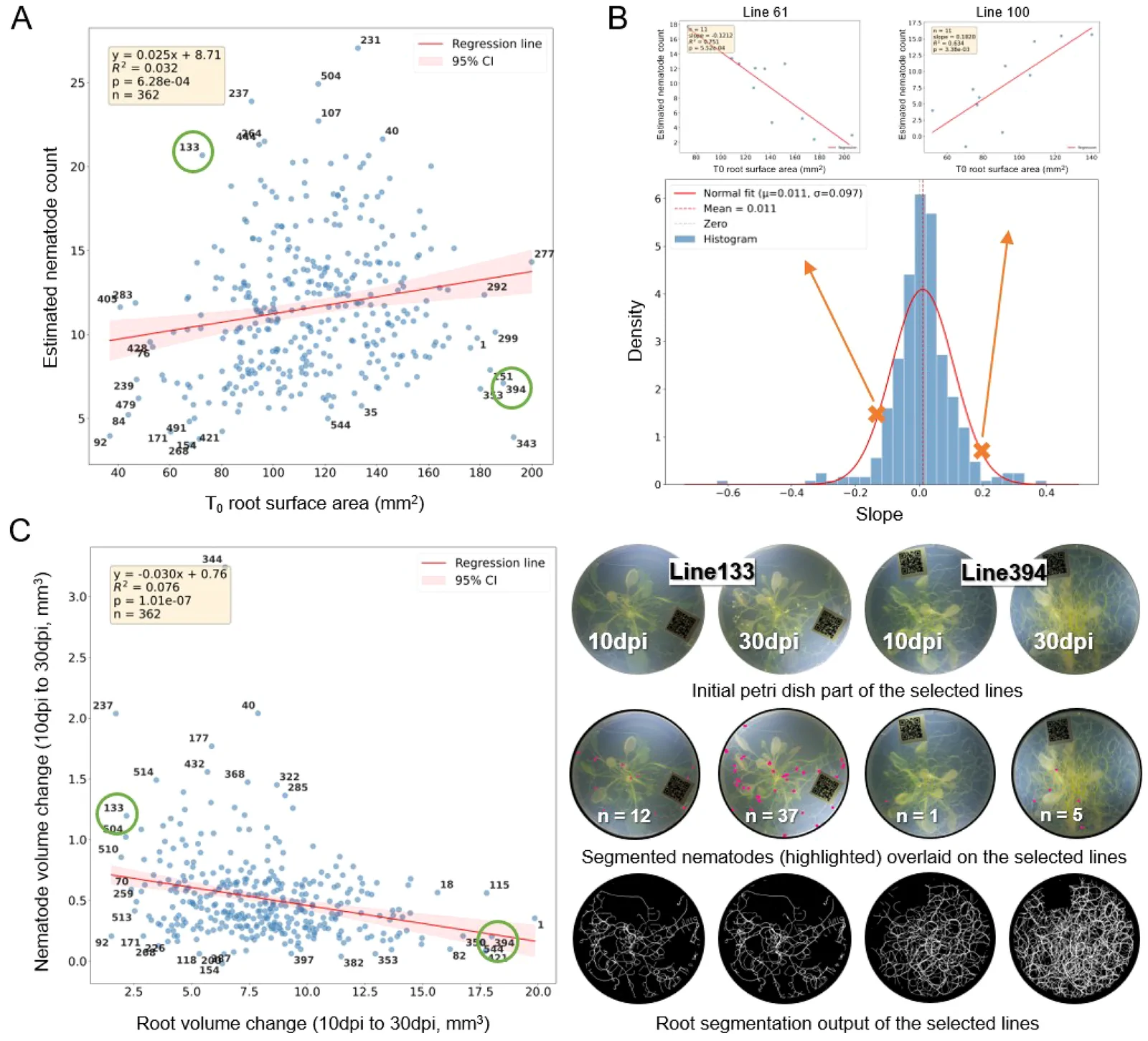
Line-level associations between root and nematode traits at establishment and reproduction stages. **(A)** Association between initial root surface area at inoculation (*T_0_*) and nematode number across 362 MAGIC lines. Each point represents a line-level phenotypic estimate derived from BLUEs. **(B)** Distribution of per-line regression slopes describing the relationship between *T_0_* root surface area and model-inferred nematode number, estimated from replicated-plate data within each line. **(C)** Relationship between net estimated root volume change and estimated nematode volume change over the 10–30 dpi (BLUEs-based line estimates). Representative lines that illustrate contrasting patterns are highlighted. The right panels show corresponding root and nematode segmentation outputs at 10 and 30 dpi.

To characterise the relationship between root system architecture and nematode reproduction across the MAGIC population, we integrated line-level phenotypic estimates derived from BLUEs for 362 genotypes (subset of 527 imaged MAGIC lines, meeting minimum data quality requirements; see Section 2.9). Two questions were addressed: 1) Is host root size at inoculation a predictor for nematode establishment?; and 2) does concurrent root expansion and nematode biomass accumulation exhibit a reciprocal trade-off during the reproductive phase? For question (1), we examined the association between initial root surface area and the (computer vision) estimated nematode count (**Figures 4A, B**); for question (2), we analysed the temporal change in root and estimated nematode volume over the 10–30 dpi infection window (**Figure 4C**).

#### 3.3.1 Linear regression approach

At the population level using linear regression, the initial root surface area (i.e. before infection) showed a minor positive association with nematode number (R^2^ = 0.032, p = 6.28 × 10^-4^, n = 362; **Figure 4A**; **Supplementary Data Sheet 1**).

The association between initial root surface area and nematode number was genotype-dependent. When these associations were estimated separately for each line from replicate-plate data, and then aggregated, their distribution was centred near zero, indicating that positive and negative per-line slope largely cancelled out at the population level (µ = 0.011, σ = 0.097; **Figure 4B**). For example, Line 100 exhibited a strongly positive per-line trend (i.e. infection increases with root surface area pre infection), whilst Line 61 showed a markedly negative trend (i.e. infection decreases with root surface area pre infection) (**Figure 4B**). In summary, across this population and under these conditions, less root area as often correlates with more nematodes, as it does with fewer nematodes.

To interrogate the longitudinal impact of, and impact on the root system, we estimated the change in estimated root volume, and the change in total estimated nematode volume, during infection. Nematode volume should provide a good proxy of parasite burden due to the metabolic demand on the host. Up until the time nematodes completed growth and thereby presumably imposes peak nutritional demand on the host (~10–30 dpi), a weak but significant negative association was observed between change in estimated root volume and change in estimated nematode volume across MAGIC lines (R^2^ = 0.076, p = 1.01 × 10^-7^, n = 362; **Figure 4C**). This pattern provides evidence of competitive resource allocation between host and parasite: lines sustaining active root growth exhibited reduced nematode biomass accumulation, whereas lines with limited root growth tended to show relatively higher parasite proliferation, consistent with a zero-sum relationship in resource use between host and parasite. Two contrasting lines illustrated the extremes of this relationship (**Figures 4C**, highlighted). Line 133, with a small initial root area (78.00 mm^2^), showed minimal root expansion between 10 and 30 dpi (10.5% growth), with estimated root volume at 110.5% of its baseline at 10 dpi (Δ = 1.33 mm^3^), whereas estimated nematode volume increased substantially, reaching 532.0% of its 10 dpi volume (Δ = 1.35 mm^3^). Segmentation results confirmed this divergence, showing little change in root structure but a marked expansion of nematode coverage (**Figure 4C**, right panels). Conversely, Line 394, with one of the largest initial root areas (195.72 mm^2^), displayed strong root expansion, reaching 188.7% of its 10 dpi volume (Δ = 24.01 mm^3^, 88.65% increase), but only a modest increase in estimated nematode volume (Δ = 0.20 mm^3^), reaching 1718.8% of an initially minute 10 dpi baseline.

#### 3.3.2 Root size classification dependent patterns

The initial root surface area and estimated nematode count exhibit a very weak, but statistically significant, correlation across the entire population (R^2^ = 0.032, p< 0.05, **Supplementary Data Sheet 1**). This global linear regression accurately captures the core relationship for the vast majority of the population, where the dynamic remains fundamentally linear. Logically, however, at the absolute lower boundary condition where host root surface area is zero, nematode infection must also be zero. Therefore the question remains: what is the critical threshold where the root surface area becomes a limiting factor?

**Figure 5:**
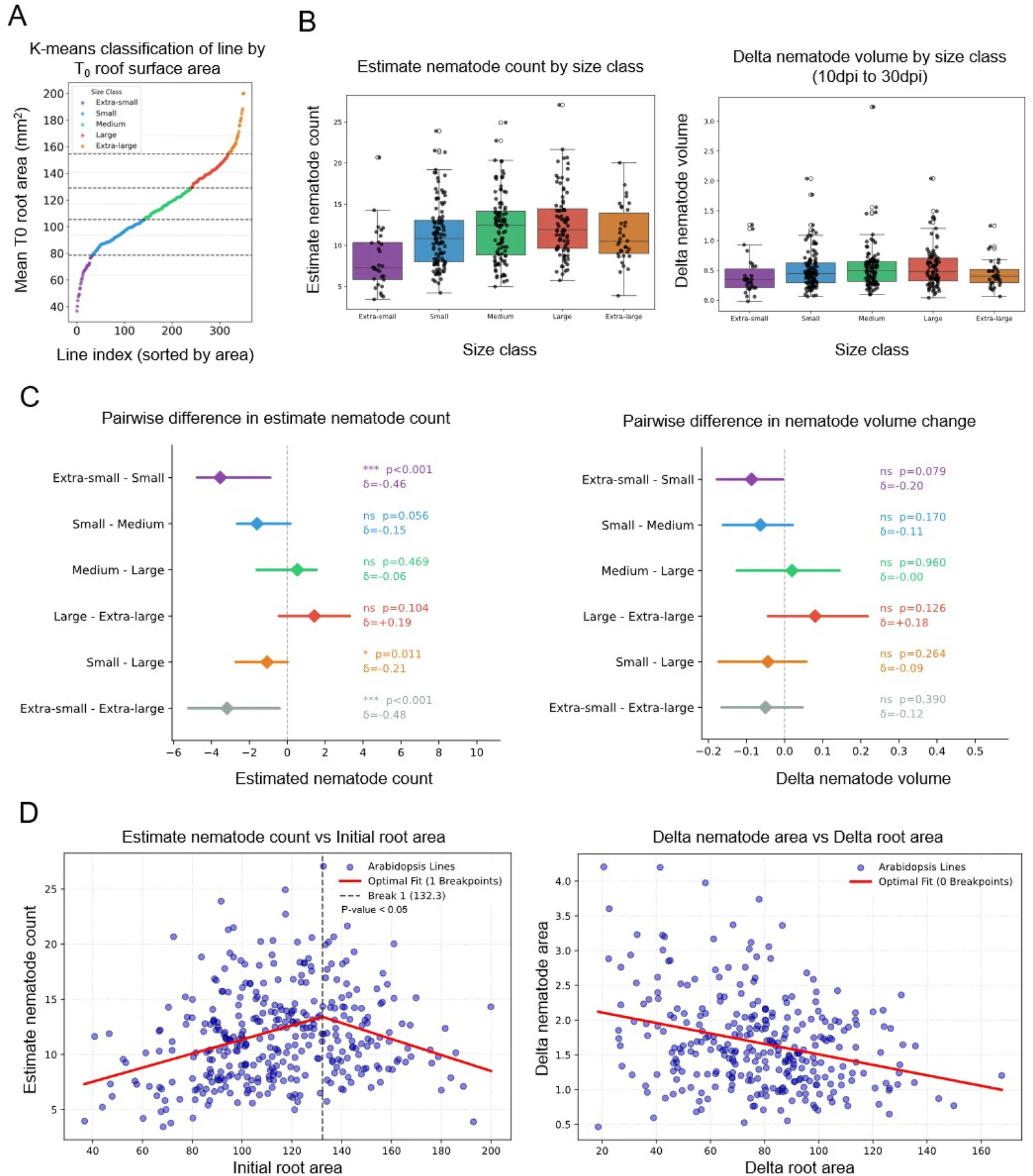
Classification of A. thaliana MAGIC population by initial root surface area. **(A)** Stratification of host resource availability. K-means clustering (k = 5) of 362 MAGIC populations based on mean *T_0_* root surface area (BLUEs). This classification establishes the baseline host resource gradient: extra-small (purple), small (blue), medium (green), large (red), and extra-large (orange). **(B)** Distribution of nematode traits across root-size classes. Boxplots show line-mean estimated nematode count (left) and estimated nematode volume change from 10 to 30 dpi (right). **(C)** Pairwise comparisons of median differences between root-size classes for estimated nematode count (left) and estimated nematode volume change (right), displayed as horizontal forest plots. Each point represents the observed difference in group medians, the bars indicate 95% bootstrap confidence intervals (5,000 resamples). Significance: ***p < 0.001, **p < 0.01, *p < 0.05, ns, not significant (Mann-Whitney U test). *δ*, Cliff’s delta effect size. **(D)** Piecewise linear regression of estimated nematode count against initial root area (left) and nematode area change against root area change from 10 to 30 dpi (right). Dashed vertical lines indicate estimated breakpoints. P-values indicate the significance of the change in slope at the breakpoint.

To investigate this we use two main approaches: 1) Binning of data; and 2) Piecewise Regression. To bin the data (1) the T0 root surface area was stratified into five distinct size classes using k-means clustering: extra-small (XS, n = 30;<77 mm^2^), small (S, n = 110; 77–104 mm^2^), medium (M, n = 104; 104–127 mm^2^), large (L, n = 84; 127–153 mm^2^), and extra-large (XL, n = 34; >153 mm^2^) (**Figure 5A**). Analysis of the stratified size classes revealed that the extra-small (XS) root group exhibited a highly significant reduction in estimated nematode number compared to all other groups (XS-S: p< 0.001, *δ* = -0.46; XS-M: p< 0.001, *δ* = -0.56; XS-L: p< 0.001, *δ* = -0.61; XS-XL: p< 0.001, *δ* = -0.48). Interestingly, estimated nematode counts did not differ significantly amongst the S, M, L, and XL groups, demonstrating that nematode infection is strictly impaired by root surface area only near the lower developmental extreme (**Figures 5B, C**, left).

Piecewise regression was utilised to achieve a more precise understanding of the true slope dynamics approaching these phenotypic extremes (**Figure 5D**). Piecewise regression shows a steeper slope in the lower end of population for nematode count in relation to root surface area than the linear approach. However, this approach also introduces an inflection point where the trend becomes negative towards higher root surface areas. In contrast to the effects observed with initial root quantity, piecewise regression analysis of dynamic change in nematode area versus change in root surface area did not reveal a significant inflection point (p > 0.05 for change in slope). This lack of a statistically supported breakpoint indicates a continuous, uniform linear correlation between these two parameters across their entire developmental spectrum. Consistent with this, no statistically significant differences in estimated nematode volume change were detected between any classes (**Figures 5B, C**, right).

## 4 Discussion

### 4.1 Automated root segmentation using the Root-TransUNet model

For *Arabidopsis thaliana* root segmentation, we developed the Root-TransUNet model, which adapted the TransUNet architecture by combining the spatial precision of a U-Net encoder-decoder with the global context awareness of ViT (Chen et al., 2021). Whilst standard CNNs often struggle with long-range dependencies in complex, highly-branched, root structures, the self-attention mechanism in Root-TransUNet maintains structural continuity across multiple scales. This capability is particularly important in the context of *Heterodera schachtii* parasitism, where root morphology undergoes progressive architectural remodelling.

In order to refine the root architecture, we designed the composite loss function to jointly address pixel-wise classification accuracy, class imbalance, and structural continuity in thin root regions. To further improve segmentation accuracy in complex infected environments, we integrated dual-stage SP modules into both the skip connections and the decoder layers. Unlike conventional square pooling kernels, which isotropically aggregate features and inadvertently incorporate agar background noise, SP modules pool along the horizontal and vertical axes to selectively capture the elongated root morphology whilst suppressing irrelevant background texture. This design substantially reduced root fragmentation in dense or overlapping regions and enabled the model to retain high-level semantic information, such as continuity of the primary root skeleton, whilst preserving low-level, fine-grained details, including subtle features of emerging lateral root tips.

Compared with traditional architectures such as UNet++ or DeepLabV3+, Root-TransUNet demonstrated superior robustness throughout the 86-day longitudinal experiment. The substantial reduction in Hd95 relative to UNet++ (0.868 vs 1.737) indicates that the improvements were not limited to pixel-level accuracy but extended to structural continuity, properties directly relevant to accurate skeleton reconstruction and trait extraction. Even under challenging imaging conditions, including leaf overlap and water vapour interference within the petri dishes, the model maintained exceptionally high axial continuity. This provides a precise and reliable foundation for phenotypic analysis, facilitating the subsequent dynamic trait analysis of host-parasite interactions.

### 4.2 Stage-dependent associations and resource allocation in host-parasite interactions

Traditional methods for assessing host susceptibility and resistance primarily involved manually counting nematode densities, female nematodes or mature cysts at specific time points (Baum et al., 2000). However, these approaches had limitations in capturing the bilateral intricacies of the infection process. By integrating our deep learning-based nematode phenotyping pipeline and the Root-TransUNet root segmentation model into hardware based digitisation methods (Wei et al. unpublished), we developed a comprehensive phenotypic framework that could resolve dynamic changes in both nematode development and root system architecture across the infection process.

#### 4.2.1 Host root architecture marginally impacts initial infection without imposing broad constraints

As demonstrated by the size-class analysis, the absolute number of established nematodes was physically constrained in exceptionally restricted root systems (extra-small class), consistent with the idea of a phenotypic bottleneck imposed by a limited availability of potential infection sites. Furthermore, a minor positive trend exists between the number of parasitizing nematodes and root surface area across the broader population. Thus, during this initial establishment phase, a smaller baseline root system exerts a subtle but detectable constraint on nematode infection numbers, indicating that the infection assay is partially bounded by host root phenotype. However, the significant phenotypic variation across the *A. thaliana* MAGIC population reveals that physical root availability alone does not dictate early establishment. This variation highlights the dominant role of host genotype in shaping basal susceptibility, wherein specific genetic backgrounds display a strong negative effect of initial root mass on infection establishment, and vice versa, averaging a near-zero impact. Taken together, in this system (5 cm petri dish with 80 J2s inoculated per plant), we can conclude that if root surface area before infection is above 77 mm^2^ then there is no rational basis to assume more root will result in more nematodes (i.e. it is not a substantive inhibitive predictor to parasitism). This allows us to disentangle the contribution of plant genotype and the physical root area on infection biology.

The extra-large root class is not statistically distinct from the broader population. However, the piecewise analysis reveals a secondary trend at the upper phenotypic extreme, where exceptionally large root surface areas are apparently negatively correlated with nematode infection. Future studies investigating these genotypes will be required to discern mechanistic insight.

#### 4.2.2 The zero sum game between nematode development and burden

Parasitism appears to follow a zero-sum linear relationship in resource use between the host and parasite, where active root growth results in reduced nematode biomass and vice versa. This antagonism manifested in distinct host-responses across the population. Some highly susceptible lines exhibited a severe developmental penalty, characterised by arrested root expansion. In contrast, tolerant hosts effectively mitigated this parasitic burden, perhaps through ongoing compensatory root growth (Willig et al., 2023). Additionally, this dynamic may reveal a nutrient distribution mechanism, controlled either by the host or the parasite, that preferentially directs resources. A possible locus for this mechanism is the physiology of the nematode feeding site. Consequently, future studies could use our findings to investigate the relationship between individual nematode growth kinetics and feeding site morphology.

Taken together, when evaluating plant resistance, it may therefore be critical to distinguish between simple initial root architectural constraints, and active physiological defences on a per genetic background basis. It also establishes a foundation for understanding resource allocation between growth and defence throughout nematode life stages and for identifying the molecular mechanisms that regulate plant-nematode interactions.

### 4.3 Limitations and further work

The Root-TransUNet deep learning-based pipeline developed in this study enabled high-throughput and automated analysis of *A. thaliana* root-related traits, and provided quantitative insights into stage-specific host-parasite interactions. Nevertheless, certain limitations still needed to be mentioned: 1) the current segmentation pipeline did not differentiate between primary and lateral roots, leaving unresolved whether host growth is driven predominantly by primary root elongation or targeted lateral root proliferation; 2) current association analyses relied on traits extracted at specific infection windows (e.g., *T_0_*, the time point of most reliable nematode detection period, and 10–30 dpi) rather than continuous dynamic traits; 3) this study focused on the underground interactions between root architecture and nematodes, lacked a coupled analysis of shoot phenotypes (e.g., leaf area, leaf greenness).

To address these limitations, the AI-powered phenotyping solution presented here is expected to evolve along several dimensions in future developments. For example: 1) future iterations of Root-TransUNet will be refined to extract the primary and lateral root, enabling investigation of whether nematodes preferentially establish on specific root orders and how this preference shapes the overall host-parasite interaction; 2) we aim to transition from specific infection window comparisons to continuous temporal traits by concurrently extracting high-resolution dynamic traits (e.g., root growth rate and nematode expansion rate) from both the host and parasite, and by registering and tracking individual roots across successive timepoints, in order to follow the developmental trajectory of each root through time and link it to local nematode establishment; 3) future frameworks may integrate coupled root phenotyping to enable simultaneous quantification of both shoot and root traits, facilitating the construction of whole-plant phenotypic models that more comprehensively capture systemic resource reallocation under parasitic stress; 4) the mechanistic basis of these extreme phenotypic trade-offs, particularly the decoupling of nematode establishment from individual biomass accumulation, remains to be resolved, and requires further experimental investigation through high-density inoculation assays on phenotypically extreme host lines. In the future, we aim to systematically integrate AI-driven, high-throughput phenotyping with multi-omics analyses (Yang et al., 2021) (e.g., transcriptomics and metabolomics) to enable precise temporal dissection of plant-parasitic nematode interactions, linking stage-specific phenotypic trajectories to their underlying molecular signatures, deepening our understanding of host-parasite resource allocation strategies, and establishing a foundation for the discovery of novel resistance mechanisms.

## Code and data availability

Python-based source code for automating root analysis using the datasets above is accessible via our GitHub repository (https://github.com/JieZhou1025/Root-nematode-interaction).

## Data Availability

Publicly available datasets were analyzed in this study. This data can be found here: https://www.ebi.ac.uk/biostudies/bioimages/studies/S-BIAD2402.

## References

[B1] Abraham N. Khan N. M. (2019). “ A novel focal Tversky loss function with improved attention U-net for lesion segmentation”, in: 2019 IEEE 16th International Symposium on Biomedical Imaging (ISBI 2019) (Venice, Italy: IEEE), 683–687.

[B2] Akintayo A. Tylka G. L. Singh A. K. Ganapathysubramanian B. Singh A. Sarkar S. (2018). A deep learning framework to discern and count microscopic nematode eggs. Sci. Rep. 8, 9145. doi: 10.1038/s41598-018-27272-w 29904135 PMC6002363

[B3] Bates D. Mächler M. Bolker B. Walker S. (2015). Fitting linear mixed-effects models Usinglme4. J. Stat. Software 67, 1–48. doi: 10.18637/jss.v067.i01

[B4] Baum T. J. Wubben M. J. Hardyy K. A. Su H. Rodermel S. R. (2000). A screen for Arabidopsis thaliana mutants with altered susceptibility to Heterodera schachtii. J. Nematol 32, 166–173. PMC2620447 19270962

[B5] Bottou L. (2010). “ Large-scale machine learning with stochastic gradient descent,” in Proceedings of COMPSTAT"2010 ( Physica-Verlag HD, Heidelberg), 177–186.

[B6] Burger W. Burge M. J. (2022). “ Scale-invariant feature transform (SIFT),” in Texts in Computer Science ( Springer International Publishing, Cham), 709–763.

[B7] Cao H. Wang Y. Chen J. Jiang D. Zhang X. Tian Q. . (2023). “ Swin-unet: Unet-like pure transformer for medical image segmentation,” in Lecture Notes in Computer Science ( Springer Nature Switzerland, Cham), 205–218.

[B8] Chen J. Lu Y. Yu Q. Luo X. Adeli E. Wang Y. . (2021). “ TransUNet: Transformers make strong encoders for medical image segmentation,” in Arxiv [Cs.CV]. Ithaca, NY, USA: arXiv (Cornell University). Available online at: http://arxiv.org/abs/2102.04306 (Accessed August 20, 2026).

[B9] Chen L. Strauch M. Daub M. Jansen M. Luigs H.-G. Merhof D. (2019). Instance segmentation of nematode cysts in microscopic images of soil samples. Annu. Int. Conf. IEEE Eng Med. Biol. Soc 2019, 5932–5936. doi: 10.1109/embc.2019.8856567 31947199

[B10] Chen L.-C. Zhu Y. Papandreou G. Schroff F. Adam H. (2018). “ Encoder-decoder with atrous separable convolution for semantic image segmentation,” in Computer Vision – Eccv 2018 ( Springer International Publishing, Cham), 833–851.

[B11] Chiu C.-L. Clack N. the napari community (2022). Napari: A python multi-dimensional image viewer platform for the research community. Microsc Microanal 28, 1576–1577. doi: 10.1017/s143192762200632841292463

[B12] Dice L. R. (1945). Measures of the amount of ecologic association between species. Ecology 26, 297–302. doi: 10.2307/1932409

[B13] Dosovitskiy A. Beyer L. Kolesnikov A. Weissenborn D. Zhai X. Unterthiner T. . (2020). An image is worth 16x16 words: Transformers for image recognition at scale. Available online at: http://arxiv.org/abs/2010.11929 (Accessed August 20, 2026).

[B14] Dutta T. K. Khan M. R. Phani V. (2019). Plant-parasitic nematode management via biofumigation using brassica and non-brassica plants: Current status and future prospects. Curr. Plant Biol. 17, 17–32. doi: 10.1016/j.cpb.2019.02.001

[B15] Fischler M. A. Bolles R. C. (1987). “ Random sample consensus: A paradigm for model fitting with applications to image analysis and automated cartography,” in Readings in Computer Vision: Issues, Problems, Principles, and Paradigms, eds Fischler M. A. Firschein O. (Los Altos, CA, USA: Morgan Kaufmann), 726–740.

[B16] Harris C. R. Millman K. J. van der Walt S. J. Gommers R. Virtanen P. Cournapeau D. . (2020). Array programming with NumPy. Nature 585, 357–362. doi: 10.1038/s41586-020-2649-2 32939066 PMC7759461

[B17] He K. Zhang X. Ren S. Sun J. (2015). Deep residual learning for image recognition. Available online at: http://arxiv.org/abs/1512.03385 (Accessed August 20, 2026).

[B18] Henderson C. R. (1975). Best linear unbiased estimation and prediction under a selection model. Biometrics 31, 423–447. doi: 10.2307/2529430 1174616

[B19] Hofmann J. Grundler F. M. W. (2006). Females and males of root-parasitic cyst nematodes induce different symplasmic connections between their syncytial feeding cells and the phloem in Arabidopsis thaliana. Plant Physiol. Biochem. 44, 430–433. doi: 10.1016/j.plaphy.2006.06.006 16889976

[B20] Hou Q. Zhang L. Cheng M.-M. Feng J. (2020). “ Strip pooling: Rethinking spatial pooling for scene parsing”, in: Proceedings of the IEEE/CVF Conference on Computer Vision and Pattern Recognition (CVPR) (Seattle, WA, USA: IEEE), 4003–4012. doi: 10.1109/cvpr42600.2020.00406

[B21] Kover P. X. Valdar W. Trakalo J. Scarcelli N. Ehrenreich I. M. Purugganan M. D. . (2009). A multiparent advanced generation inter-cross to fine-map quantitative traits in Arabidopsis thaliana. PloS Genet. 5, e1000551. doi: 10.1371/journal.pgen.1000551 19593375 PMC2700969

[B22] Kranse O. P. Ko I. Healey R. Sonawala U. Wei S. Senatori B. . (2022). A low-cost and open-source solution to automate imaging and analysis of cyst nematode infection assays for Arabidopsis thaliana. Plant Methods 18, 134. doi: 10.1186/s13007-022-00963-2 36503537 PMC9743603

[B23] Kuijken R. C. P. van Eeuwijk F. A. Marcelis L. F. M. Bouwmeester H. J. (2015). Root phenotyping: from component trait in the lab to breeding. J. Exp. Bot. 66, 5389–5401. doi: 10.1093/jxb/erv239 26071534

[B24] Kuznetsova A. Brockhoff P. B. Christensen R. H. B. (2017). LmerTest package: Tests in linear mixed effects models. J. Stat. Software 82, 1–26. doi: 10.18637/jss.v082.i13

[B25] Likas A. Vlassis N. Verbeek J. (2003). The global k-means clustering algorithm. Pattern Recognit. 36, 451–461. doi: 10.1016/S0031-3203(02)00060-225079929

[B26] Macbeth G. Razumiejczyk E. (2011). Rubén Daniel Ledesma. Cliff’s Delta Calculator: non-parametric effect size program two groups observations. Universitas Psychologica 10, 545–555. doi: 10.11144/Javeriana.upsy10-2.cdcp

[B27] McKinney W. (2010). “ Data Structures for Statistical Computing in Python”, in: Proceedings of the 9th Python in Science Conference (Austin, TX, USA: SciPy), 56–61.

[B28] Nachar N. (2008). The Mann-Whitney U: A test for assessing whether two independent samples come from the same distribution. Tutor Quant Methods Psychol. 4, 13–20. doi: 10.20982/tqmp.04.1.p013

[B29] Narisetti N. Henke M. Seiler C. Junker A. Ostermann J. Altmann T. . (2021). Fully-automated root image analysis (faRIA). Sci. Rep. 11, 16047. doi: 10.1038/s41598-021-95480-y 34362967 PMC8346561

[B30] O’Shea K. Nash R. (2015). An introduction to convolutional neural networks. arXiv [cs.NE]. arXiv:1511.08458. doi: 10.48550/ARXIV.1511.08458

[B31] Paez-Garcia A. Motes C. M. Scheible W.-R. Chen R. Blancaflor E. B. Monteros M. J. (2015). Root traits and phenotyping strategies for plant improvement. Plants 4, 334–355. doi: 10.3390/plants4020334 27135332 PMC4844329

[B32] Paszke A. Gross S. Massa F. Lerer A. Bradbury J. Chanan G. . (2019). PyTorch: An imperative style, high-performance deep learning library. Available online at: http://arxiv.org/abs/1912.01703 (Accessed August 20, 2026).

[B33] Pedregosa F. Varoquaux G. Gramfort A. Michel V. Thirion B. Grisel O. . (2012). Scikit-learn: Machine learning in Python. Available online at: http://arxiv.org/abs/1201.0490 (Accessed August 20, 2026).

[B34] Pei S.-C. Horng J.-H. (1995). Circular arc detection based on Hough transform. Pattern Recognit. Lett. 16, 615–625. doi: 10.1016/0167-8655(95)80007-g

[B35] Pilgrim C. (2021). piecewise-regression (aka segmented regression) in Python. J. Open Source Softw. 6 (68). doi: 10.21105/joss.03859

[B36] Ronneberger O. Fischer P. Brox T. (2015). U-Net: Convolutional networks for biomedical image segmentation. Available online at: http://arxiv.org/abs/1505.04597 (Accessed August 20, 2026).

[B37] Russell V. L. Piaskowski J. (2026). emmeans: Estimated Marginal Means, aka Least-Squares Means. Available online at: https://rvlenth.github.io/emmeans/ (Accessed August 20, 2026).

[B38] Russell B. C. Torralba A. Murphy K. P. Freeman W. T. (2008). LabelMe: A database and web-based tool for image annotation. Int. J. Comput. Vis. 77, 157–173. doi: 10.1007/s11263-007-0090-830311153

[B39] Saikai K. K. Bresilla T. Kool J. de Ruijter N. C. A. van Schaik C. Teklu M. G. (2024). Counting nematodes made easy: leveraging AI-powered automation for enhanced efficiency and precision. Front. Plant Sci. 15, 1349209. doi: 10.3389/fpls.2024.1349209 38993936 PMC11238600

[B40] Seidenthal K. Panjvani K. Chandnani R. Kochian L. Eramian M. (2022). Iterative image segmentation of plant roots for high-throughput phenotyping. Sci. Rep. 12, 16563. doi: 10.1038/s41598-022-19754-9 36195610 PMC9532414

[B41] Shapiro S. S. Wilk M. B. (1965). An analysis of variance test for normality (complete samples). Biometrika 52 (3–4), 591–611.

[B42] Shit S. Paetzold J. C. Sekuboyina A. Ezhov I. Unger A. Zhylka A. . (2021). “ ClDice - a novel topology-preserving loss function for tubular structure segmentation”, in: Proceedings of the IEEE/CVF Conference on Computer Vision and Pattern Recognition (CVPR) (Nashville, TN, USA: IEEE), 16560–16569. doi: 10.1109/cvpr46437.2021.01629

[B43] Sijmons P. C. Grundler F. M. W. von Mende N. Burrows P. R. Wyss U. (1991). Arabidopsis thaliana as a new model host for plant‐parasitic nematodes. Plant J. 1, 245–254. doi: 10.1111/j.1365-313X.1991.00245.x37945311

[B44] Smith A. G. Petersen J. Selvan R. Rasmussen C. R. (2020). Segmentation of roots in soil with U-Net. Plant Methods 16, 13. doi: 10.1186/s13007-020-0563-0 32055251 PMC7007677

[B45] Sun G. Feng F. Wang D. Ju L. Xu X. Yan M. . (2026). BloomSight: An ultra-high-frequency phenotyping framework for diurnal flowering dynamics in japonica and indica rice to enable genetic dissection and hybrid-breeding applications. Plant Phenomics 8, 100215. doi: 10.1016/j.plaphe.2026.100215 42087926 PMC13137185

[B46] Sun G. Lu H. Zhao Y. Zhou J. Jackson R. Wang Y. . (2022). AirMeasurer: open-source software to quantify static and dynamic traits derived from multiseason aerial phenotyping to empower genetic mapping studies in rice. New Phytol. 236, 1584–1604. doi: 10.1111/nph.18314 35901246 PMC9796158

[B47] Virtanen P. Gommers R. Oliphant T. E. Haberland M. Reddy T. Cournapeau D. . (2020). SciPy 1.0: fundamental algorithms for scientific computing in Python. Nat. Methods 17, 261–272. doi: 10.1038/s41592-019-0686-2 32015543 PMC7056644

[B48] Willig J.-J. Sonneveld D. van Steenbrugge J. J. M. Deurhof L. van Schaik C. C. Teklu M. G. . (2023). From root to shoot: quantifying nematode tolerance in Arabidopsis thaliana by high-throughput phenotyping of plant development. J. Exp. Bot. 74, 5487–5499. doi: 10.1093/jxb/erad266 37432651 PMC10540735

[B49] Yang Y. Saand M. A. Huang L. Abdelaal W. B. Zhang J. Wu Y. . (2021). Applications of multi-omics technologies for crop improvement. Front. Plant Sci. 12, 563953. doi: 10.3389/fpls.2021.563953 34539683 PMC8446515

[B50] Yasrab R. Atkinson J. A. Wells D. M. French A. P. Pridmore T. P. Pound M. P. (2019). RootNav 2.0: Deep learning for automatic navigation of complex plant root architectures. GigaScience 8, giz123. doi: 10.1093/gigascience/giz123 31702012 PMC6839032

[B51] Zhou Z. Siddiquee M. M. R. Tajbakhsh N. Liang J. (2018). UNet++: A nested U-Net architecture for medical image segmentation. Available online at: http://arxiv.org/abs/1807.10165 (Accessed August 20, 2026). 10.1007/978-3-030-00889-5_1 PMC7329239 32613207

