# Supplementary material for: Root-TransUNet enables high-throughput phenotyping of Arabidopsis thaliana roots as a parameter in Heterodera schachtii parasitism
Source: Front Plant Sci. 2026 Sep 4;17:1939674. doi: 10.3389/fpls.2026.1939674 (PMC13585716; doi:10.3389/fpls.2026.1939674)
Supplement: Supplementary file 1 [file DataSheet1.pdf]

Supplementary Material

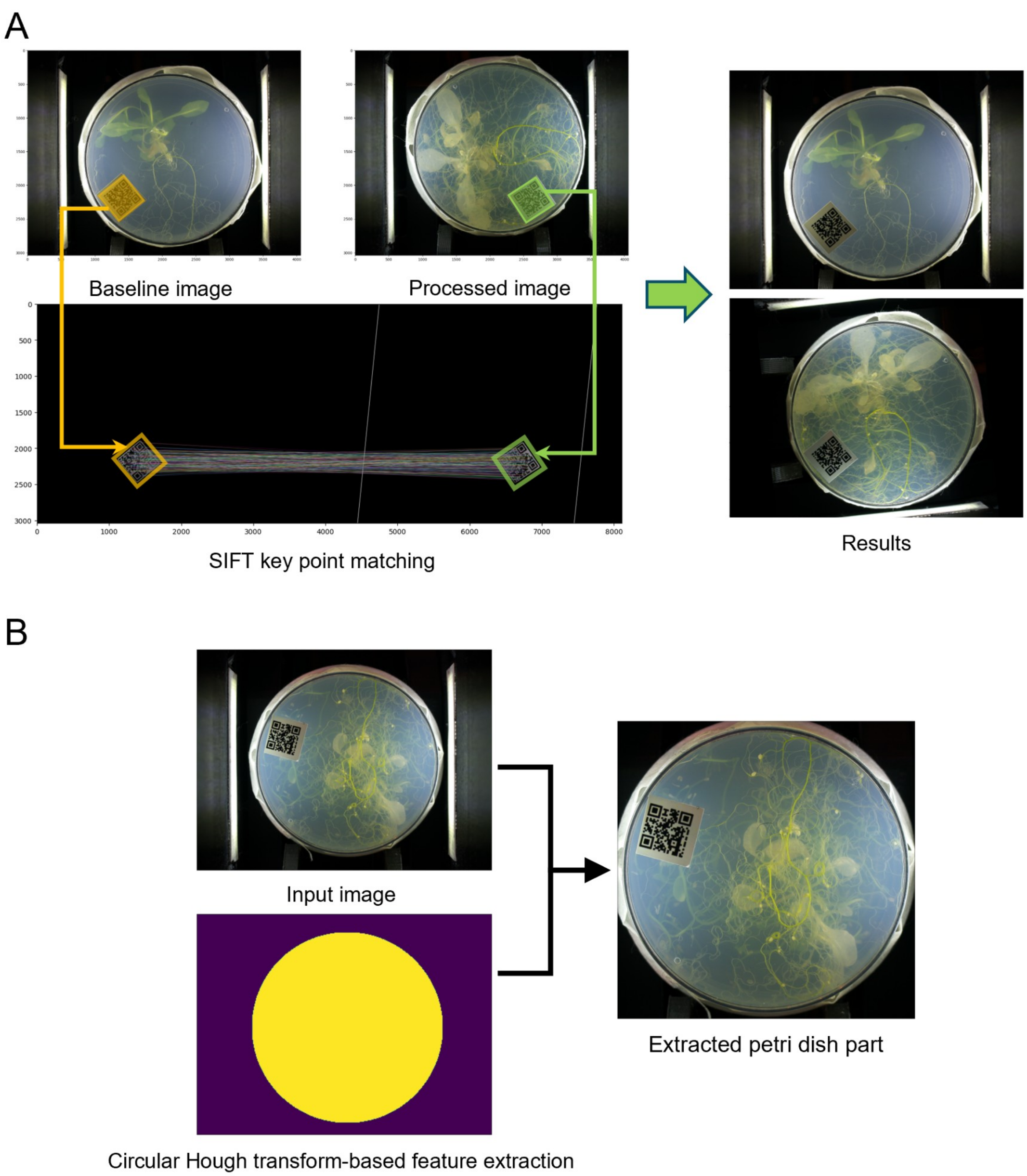

Figure S1. Image pre-processing pipeline. A) Spatial registration employs SIFT-based keypoint matching. A baseline image (orange frame) and a target image (green frame) align by matching keypoints associated with the QR-code marker embedded in each petri dish (expanded panel). RANSAC-based homography estimation corrects geometric distortions, resulting in spatially registered images across time points**. B)** Extraction of the petri dish region using the circular Hough transform. A standardised circular mask (yellow) was generated from the registered image and applied to accurately crop the interior of the petri dish.

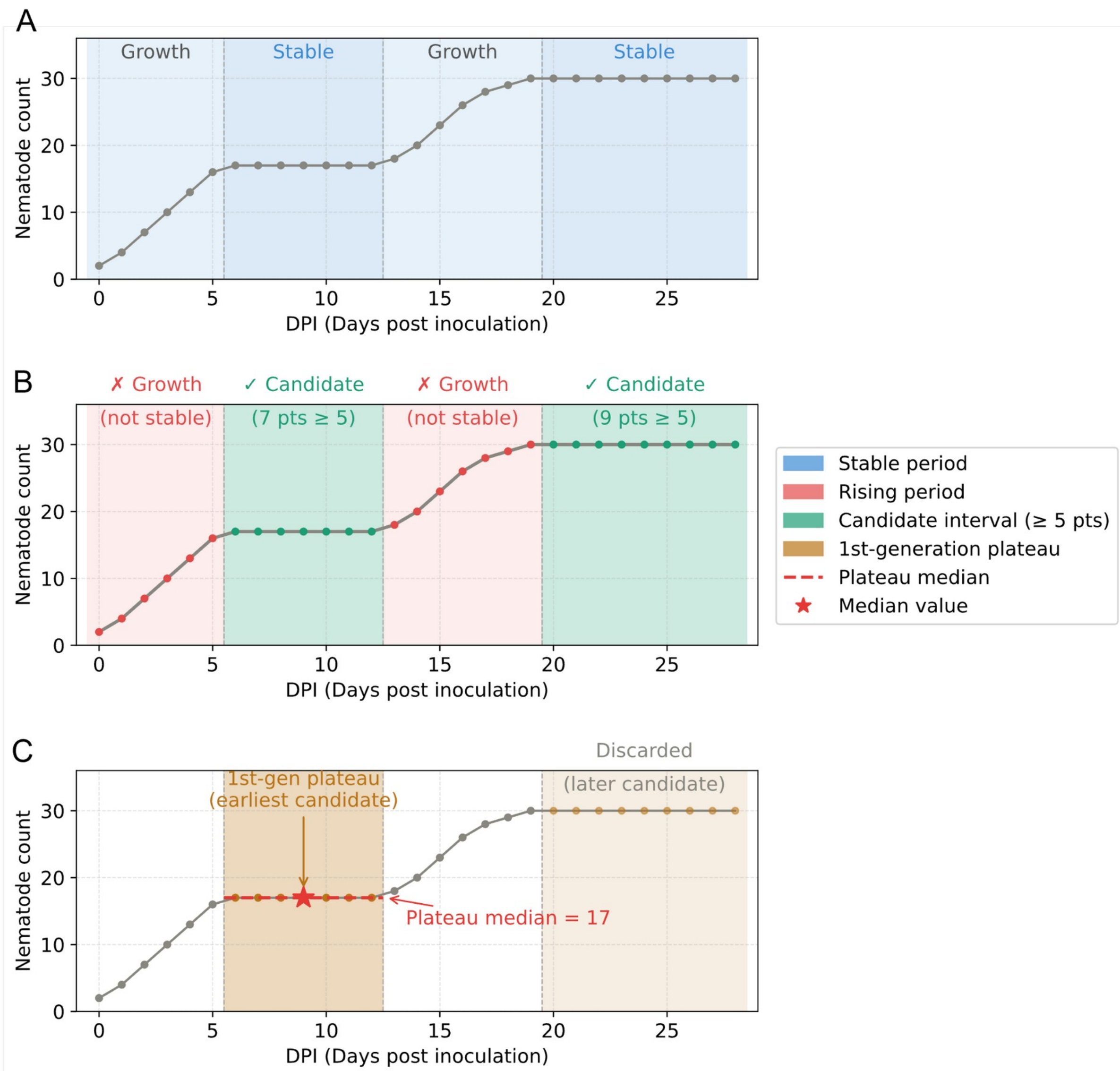

Figure S2. Schematic illustration of the data-driven algorithm for detecting the first-generation plateau in nematode population dynamics. A) The population curve is segmented into consecutive intervals of either growth or stability. Stable intervals (blue shading) represent periods during which nematode abundance remained effectively constant across successive imaging sessions, whereas growth intervals (unshaded) correspond to periods of active population increase. **B)** Candidate stable intervals are extracted by applying a minimum duration criterion: only intervals spanning at least five consecutive imaging time points are retained (√ Candidate; green shading), while shorter intervals or those failing the stability criterion are excluded (**✗** Growth; red shading). **C)** The retained candidate intervals are sorted chronologically, and the earliest qualifying interval is designated as the first-generation plateau (amber shading). The representative plateau trait is defined as the median nematode count within this interval (red dashed line; ★), providing a robust estimate of the mature adult female population at the completion of the first infection cycle.

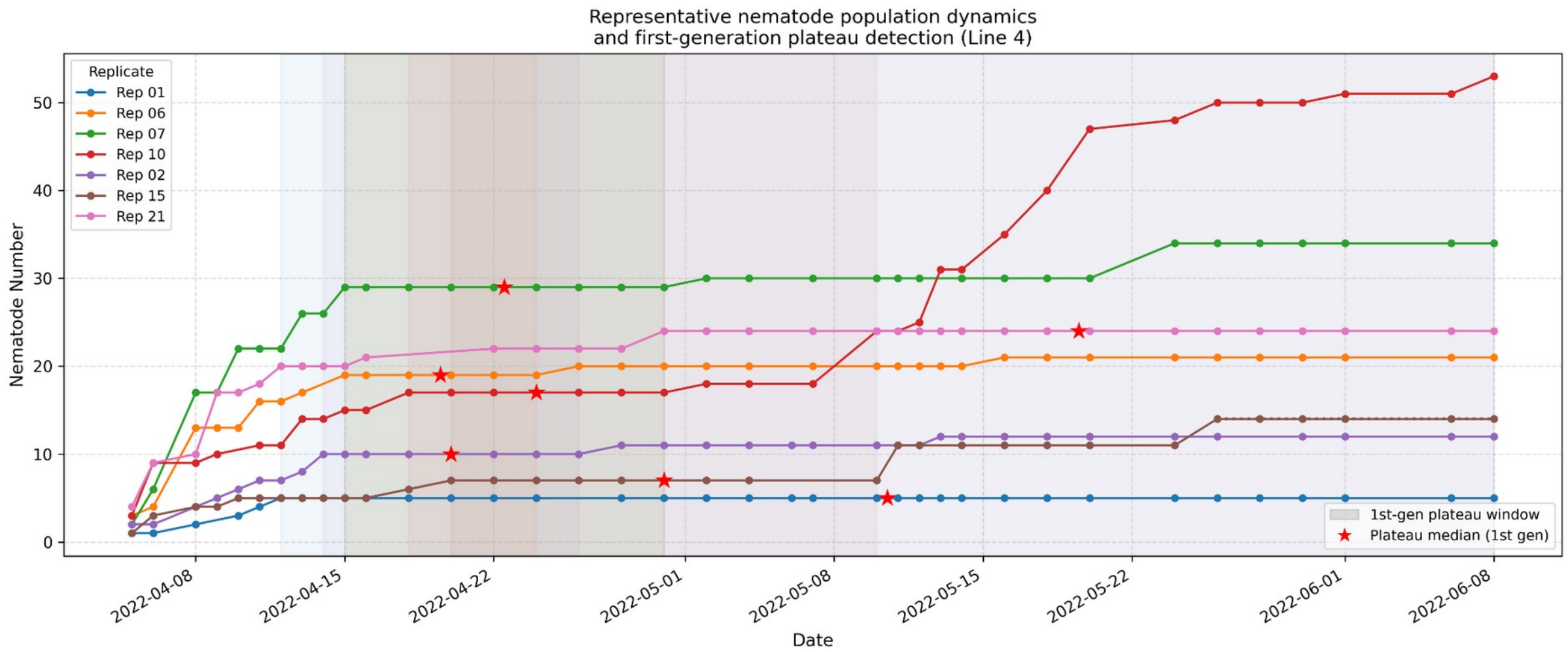

Figure S3. Representative nematode population dynamics illustrating the detection of a first-generation plateau in a single *A. thaliana* MAGIC line. Each coloured line represents the trajectory of estimated nematode counts for an individual replicate plate throughout the 86-day imaging period (n = 7). Shaded colour regions indicate the first-generation plateau windows identified for each replicate. A red star (★) marks the median estimated nematode count within each identified plateau window.

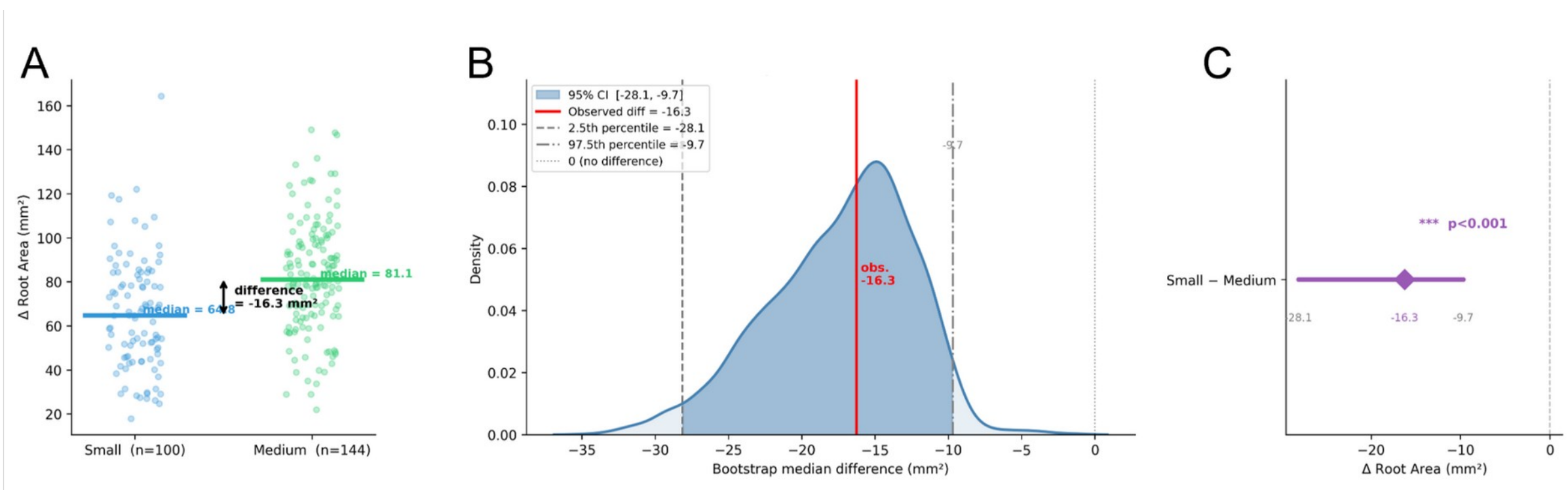

Figure S4. Schematic overview of the statistical framework used for pairwise comparisons of trait changes across root size classes. A) Strip plot of line-level trait changes (Δ) in each class; horizontal lines indicate the group medians and their observed difference. **B)** Bootstrap distribution of the median difference (5,000 resamples with replacement): the red line indicates the observed difference; the dashed lines indicate the 2.5th and 97.5th percentiles (95% confidence interval); and the dotted line indicates zero. **C)** Forest plot showing the observed median difference (diamond) and its 95% bootstrap confidence interval (horizontal bar); intervals not crossing zero indicate a statistically significant difference. Statistical significance was assessed using the Mann-Whitney U test (*** $p < 0.001$, ** $p < 0.01$, * $p < 0.05$, ns = not significant), and effect size was quantified using Cliff's delta (δ; range: −1 to +1).

Subset	Number	Days post-inoculation range
Training	998	9-63
Validation	125	9-63
Test	125	9-63

Table S1. Summary of the annotated dataset used for Root-TransUNet training, validation, and testing.

Model	Dice	IoU	Precision	Recall	Hd95
TransUNet	0.935	0.879	0.935	0.930	1.176
TransUNet + composite loss	0.937	0.884	0.950	0.926	1.021

Table S2. Effect of the composite loss function on the segmentation performance of the baseline TransUNet

SP module			Dice	IoU	Precision	Recall
Encoder	Skip-connection	Decoder				
×	×	×	0.937	0.884	0.950	0.926
√	×	×	0.938	0.883	0.936	0.934
×	√	×	0.940	0.891	0.935	0.932
×	×	√	0.946	0.897	0.944	0.942
√	√	×	0.948	0.899	0.942	0.945
×	√	√	0.949	0.902	0.941	0.955

Table S3. Ablation study of Strip Pooling (SP) module configurations.

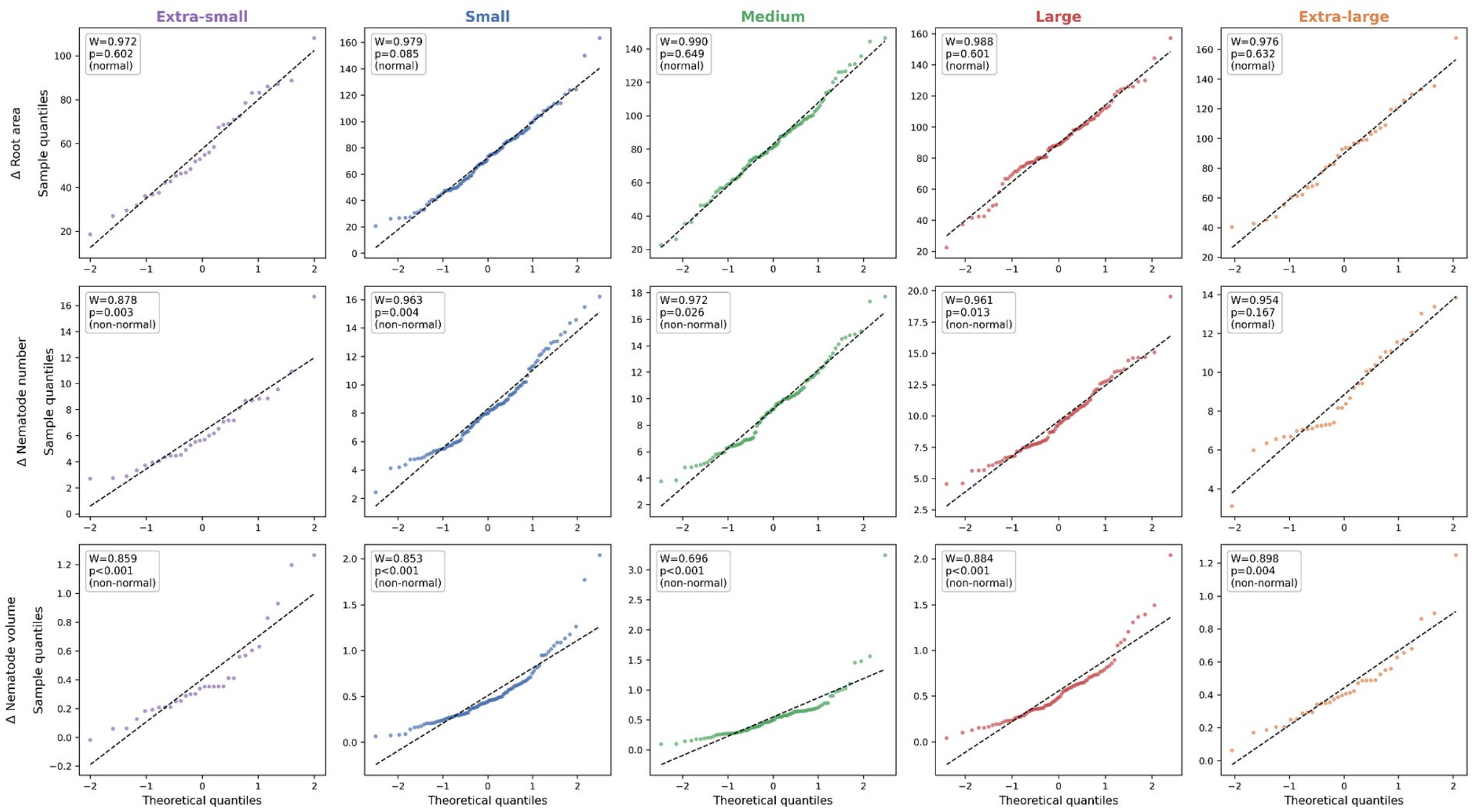

Figure S5. Normality assessment of trait-change distributions across root size classes. Quantile-quantile (Q-Q) plots of line-level trait changes (Δ, 10-30 dpi) for Δ root area, Δ nematode number and Δ nematode volume (rows), across the five K-means size classes (columns: Extra-small, Small, Medium, Large, Extra-large).

This is a large table, this is attached as a separate csv file.

Supplementary dataset 1. Summary table of nematode and root traits BLUEs by line.
